# Brown Adipose Tissue Harbors a Distinct Sub-Population of Regulatory T Cells

**DOI:** 10.1371/journal.pone.0118534

**Published:** 2015-02-25

**Authors:** Dasa Medrikova, Tjeerd P. Sijmonsma, Katharina Sowodniok, David M. Richards, Michael Delacher, Carsten Sticht, Norbert Gretz, Tobias Schafmeier, Markus Feuerer, Stephan Herzig

**Affiliations:** 1 Joint Research Division Molecular Metabolic Control, German Cancer Research Center (DKFZ) Heidelberg, Center for Molecular Biology (ZMBH) and University Hospital, Heidelberg University, 69120, Heidelberg, Germany; 2 Institute for Diabetes and Cancer IDC, Helmholtz Center Munich, 85764 Neuherberg, and Joint Heidelberg-IDC Translational Diabetes Program, Heidelberg University Hospital, 69120, Heidelberg, Germany; 3 Helmholtz Young Investigator Research Group Immune Tolerance, German Cancer Research Center (DKFZ) Heidelberg, 69120, Heidelberg, Germany; 4 Center for Medical Research, University Clinics Mannheim, 68167, Mannheim, Germany; Jackson Laboratory, UNITED STATES

## Abstract

Regulatory T (T_reg_) cells are critical determinants of both immune responses and metabolic control. Here we show that systemic ablation of T_reg_ cells compromised the adaptation of whole-body energy expenditure to cold exposure, correlating with impairment in thermogenic marker gene expression and massive invasion of pro-inflammatory macrophages in brown adipose tissue (BAT). Indeed, BAT harbored a unique sub-set of T_reg_ cells characterized by a unique gene signature. As these T_reg_ cells respond to BAT activation upon cold exposure, this study defines a BAT-specific T_reg_ sub-set with direct implications for the regulation of energy homeostasis in response to environmental stress.

## Introduction

Obesity has reached pandemic dimensions with more than 1.5 billion people being affected world-wide [1]. Nutritional overloading of triglyceride storing white adipose tissue (WAT) impairs tissue function and is associated with a status of chronic low-grade inflammation [2]. Upon energy excess, adipocytes release chemokines resulting in adipose tissue infiltration by innate immune cells, e.g. pro-inflammatory macrophages and mast cells, and consequently aberrantly elevated inflammatory signaling [3]. Of note, WAT inflammation represents a causative factor for insulin resistance as a hallmark of obesity-related metabolic dysfunction [4]. Consequently, anti-inflammatory treatment potently improves insulin sensitivity in obesity [5]. In addition to the well-established role of the innate immune system, i.e. macrophage infiltration, in WAT dysfunction and a shift in macrophage polarization from an anti-inflammatory to a more pro-inflammatory status during progressive adiposity, the (aberrant) function of adaptive immune cells is increasingly emerging as a key event in obesity-related metabolic complications [6].

In this respect, regulatory T (T_reg_) cells represent a diverse sub-population of CD4^+^ T cells characterized by specific expression of the forkhead-winged helix transcription factor Foxp3 [7]. T_reg_ cells interact with components of both the innate and the adaptive immune system, thereby serving as negative feedback regulators which prevent excess immune responses and ensure self-tolerance [8]. Whereas distinct sub-sets of T lymphocytes, like pro-inflammatory CD4^+^ T-helper (T_H_1) cells and cytotoxic T cells, were shown to be upregulated in obese WAT and might also contribute to insulin resistance [9], the number of WAT T_reg_ cells was found to be markedly reduced in obese mice and humans [7,10]. Furthermore, transfer of T_reg_ cells into lymphocyte-deficient obese mice reversed the aberrant glucose metabolism of the animals [11], indicating a key role of T_reg_ cells in controlling WAT inflammation and the associated insulin sensitivity. Importantly, visceral adipose tissue (VAT) T_reg_ cell accumulation, phenotype and function are controlled by the transcription regulator peroxisome proliferator activated receptor gamma (Pparg) [12].

In contrast to the energy-storing WAT, brown adipose tissue (BAT) and inducible brown-in-white (brite) adipocytes are specialized in the dissipation of energy in the form of heat by so-called uncoupling thermogenesis, mediated by the dissociation of mitochondrial respiratory chain electron transport from ATP synthesis via the action of uncoupling protein (UCP)1 [13]. Singular studies indicated that immune cells may also exert important regulatory roles in BAT development and physiology. For instance, alternatively activated anti-inflammatory macrophages (AAM) have been detected in WAT and BAT of mice in response to cold stimulation [14]. Moreover, AAM produced and secreted noradrenaline in an IL-4-dependent manner, thereby increasing thermogenic gene expression in BAT and enhancing energy expenditure [14]. In addition, AAM appear to be centrally involved in WAT browning, i.e. the appearance of brite cells, in response to beta-adrenergic signaling [15]. Recent studies reported that adipose tissue-resident eosinophils induced browning of WAT by stimulating AAM-dependent catecholamine release [16]. Finally, mice lacking mast cells display enhanced energy expenditure, improved glucose homeostasis and elevated expression of UCP1 in BAT [17]. Functional BAT has been detected and implicated in obesity susceptibility in adult humans [18]. Thus, the modulation of BAT-specific immune cell functions may provide future opportunities for BAT-centered systemic control of energy homeostasis and therapeutic targeting of obesity-related metabolic dysfunction. However, the regulatory impact and the molecular nature of BAT-associated T_reg_ cells have not been defined to date.

## Methods

### 2.1 Animals

**T**_**reg**_ **cells isolation**. C57Bl6 female mice (n = 120) were obtained from Charles River Laboratories (CRL) at age 8 weeks and used for isolation of T_reg_ cells from BAT. Mice were housed in a temperature controlled cabinet (Memmert) on a 12h light-dark cycle with unrestricted access to food and water. Mice were acclimatized to 30°C for 2 weeks and afterwards half of them (n = 60) was sacrificed and used for isolation of “warm” T_reg_ cells. The other half (n = 60) was subjected to 12°C cold challenge for 2 days, sacrificed and used for isolation of “cold” T_reg_ cells (details of T_reg_ cells isolation below).

**Metabolic phenotyping of T_reg_-depleted mice**. B6N.129(Cg)-Foxp3^tm3Ayr^ (Foxp3DTR; MGI:3698131; Jackson Stock Number: 016958) mice [19] were bred in our specific pathogen free facility at the DKFZ. The mice were housed at ambient temperature 22°C and fed standard chow diet (Kliba Nafag #3437, Provimi Kliba). For metabolic phenotyping of T_reg_ cell-depleted mice, FoxP3DTR female mice were housed individually with unrestricted access to food and water in a control environment on 12h light-dark cycle in the PhenoMaster Cage System (TSE Systems) [20]. In order to deplete T_reg_ cells, mice (n = 10) were intraperitoneally injected with 40 ng per g of body weight diphtheria toxin (DT) (Sigma) diluted in 200 μL PBS on two consecutive days. Vehicle (PBS) injected mice (n = 10) served as control. After the first round of injections, the housing temperature was decreased to 12°C. Oxygen consumption, food intake, locomotor activity and body weight (BW) were recorded continuously for 4 days. Heat production was calculated according to the formula: Heat production = CV*VO_2_, where CV = 3.941+(1.106*Respiratory Exchange Ratio) wherein numerical values are computed by TSE system. Mice were sacrificed with cervical dislocation in a random-fed state. Serum, brown adipose tissue, inguinal white adipose tissue and spleen were collected, washed in PBS, snap-frozen in liquid nitrogen and stored at -80°C. Animal handling and experimentation was performed in accordance with the European Union directives and the German animal welfare act (Tierschutzgesetz) and approved by local authorities (Regierungspräsidium Karlsruhe).

### 2.2 Cell sorting

For microarray analysis of T_reg_ cells, stromo-vascular fraction from brown adipose tissue of warm acclimated mice (30°C for 2 weeks), cold treated mice (12°C for 2 days) and spleen was isolated and T_reg_ cells were sorted out using fluorescence-activated cell sorting (FACS). Nonspecific binding of antibodies to cells was blocked by incubation with rat and hamster serum (1.0% (vol/vol) each) and cells were labeled with the following antibodies (clone): CD4 (GK1.5), CD8α (53–6.7), CD19 (6D5), TCRβ (H57–597), CD25 (PC61), CD11b (M1/70) and CD45 (30-F11) (Biolegend). Cells were FACS purified with the FACSAria II instrument (BD Bioscience). Antibody quality was checked and gating was performed using isotype controls. Purity of cell sorts was assured through post-sort analysis and post-sort staining. FlowJo (Treestar) was used for the analysis of flow cytometry data.

### 2.3 RNA expression profiling of FACS-sorted T cell populations

Conventional T cell (T_conv_) (CD4+CD25-) or regulatory T cell (T_reg_) (CD4+CD25+) populations were FACS purified from the spleen or brown adipose tissue (BAT) from warm (30°C) or cold (12°C) treated mice. RNA was extracted using the RNEasy Plus Micro Kit (Qiagen). The DKFZ Genomics and Proteomics Core Facility amplified and hybridized material to the MouseWG-6 v2.0 Expression BeadChip (Illumina). Microarray data have been deposited in the Gene Expression Omnibus (GEO) with the accession code GSE64909. Microarray confirmation by real-time PCR was not performed for T_reg_ cells due to sample amount limitations.

### 2.4 Blood and serum parameters

Blood glucose was measured at random-fed state immediately after sacrificing the mice using an automated glucose monitor (One Touch, Lifescan). Commercially available kits were used to quantify serum triglycerides (Sigma) and non-esterified fatty acids (WAKO).

### 2.5 RNA isolation and real-time RT-PCR

Total RNA was extracted from frozen powderized BAT, scWAT and spleen using Qiazol (Qiagen) and the RNeasy Mini Kit (Qiagen), performing on-column DNAse digest. cDNA was prepared by reverse transcription of 1 μg of total RNA using the First strand cDNA synthesis kit (Fermentas). Real-time RT-PCR was performed in order to measure gene expression of browning (Ucp1, Cidea, Dio2, Pparg, Prdm16) and inflammatory (Cd68, Ccl2, Tnfa, Ifng, Mrc1, Mgl1, Arg1, Il-10, Il-4) markers in BAT and scWAT and to verify T_reg_ cells depletion from BAT and spleen by measuring FoxP3 expression. TaqMan Master Mix (Applied Biosystems) and a gene specific Taqman probes (Applied Biosystems) were used on an ABI StepOnePlus sequence detector (Applied Biosystems). Relative mRNA expression levels were calculated by the delta Ct method using TATA-box binding protein (Tbp) expression as a reference.

### 2.6 RNA expression profiling of T_reg_-deficient and -proficient BAT

Total RNA from BAT was isolated as described and subjected to gene expression analysis using GeneChip Mouse Genome 430 2.0 arrays (Affymetrix) performed according to the manufacturer’s recommendations. A Custom CDF Version 14 with Entrez based gene definitions was used to annotate the arrays. The Raw fluorescence intensity values were normalized by quantile normalization. Differential gene expression was analyzed based on loglinear mixed model ANOVA with a commercial software package SAS JMP7 Genomics (version 4) from SAS (SAS Institute). Significance was assumed based on a false positive rate of p = 0.05 with FDR correction. Gene Set Enrichment Analysis (GSEA), was used to determine whether defined lists (or sets) of genes exhibit a statistically significant bias in their distribution within a ranked gene list (http://www.broadinstitute.org/gsea/; [21]. Raw and normalized data are deposited in the Gene Expression Omnibus (GEO) database with an accession number GSE62157.

Real-time PCR was performed on six selected genes, including Prdm16, Dio2, Cd68, Ccl2, Il4, and Infγ to confirm the array results.

### 2.7 Histology

Brown adipose tissue was fixed in 4% paraformaldehyde for 24 h at room temperature, paraffin-embedded and cut in 3 μm sections. Sections were stained with hematoxylin and eosin (H&E) by standard procedures. For immunohistochemistry, sections were subjected to heat-mediated antigen retrieval in citrate buffer (pH 6) prior to blocking with 1% BSA for 1 h at room temperature. Primary rat monoclonal anti-MAC-2 antibody (Cedarlane Laboratories, #CL8942AP) was diluted 1:3800 in 1% BSA and incubated over night at 4°C. For detection, HPR-conjugated goat anti-rat antibody (Santa Cruz Biotechnology, #sc-2032) diluted 1:500 in 1% BSA was used as a secondary antibody with 3,3’-diaminobenzidine (Sigma) as a chromogenic substrate. Stainings were imaged at 20x magnification (Zeiss). MAC-2 positive signal was quantified as a percentage of total area using Fiji software [22].

### 2.8 Statistical Analysis

Statistical analysis of experimental data was performed using Prism software (GraphPad) or Excel (Microsoft). The results are shown as means ± SD. Statistical analysis was performed by two-tailed Student’s t-test, for real-time RT-PCR calculated on log10-transformed data values. Results were considered statistically significant if p-values were less than 0.05.

## Results

### Cold BAT T_reg_ cells display a unique molecular signature

**Fig 1:**
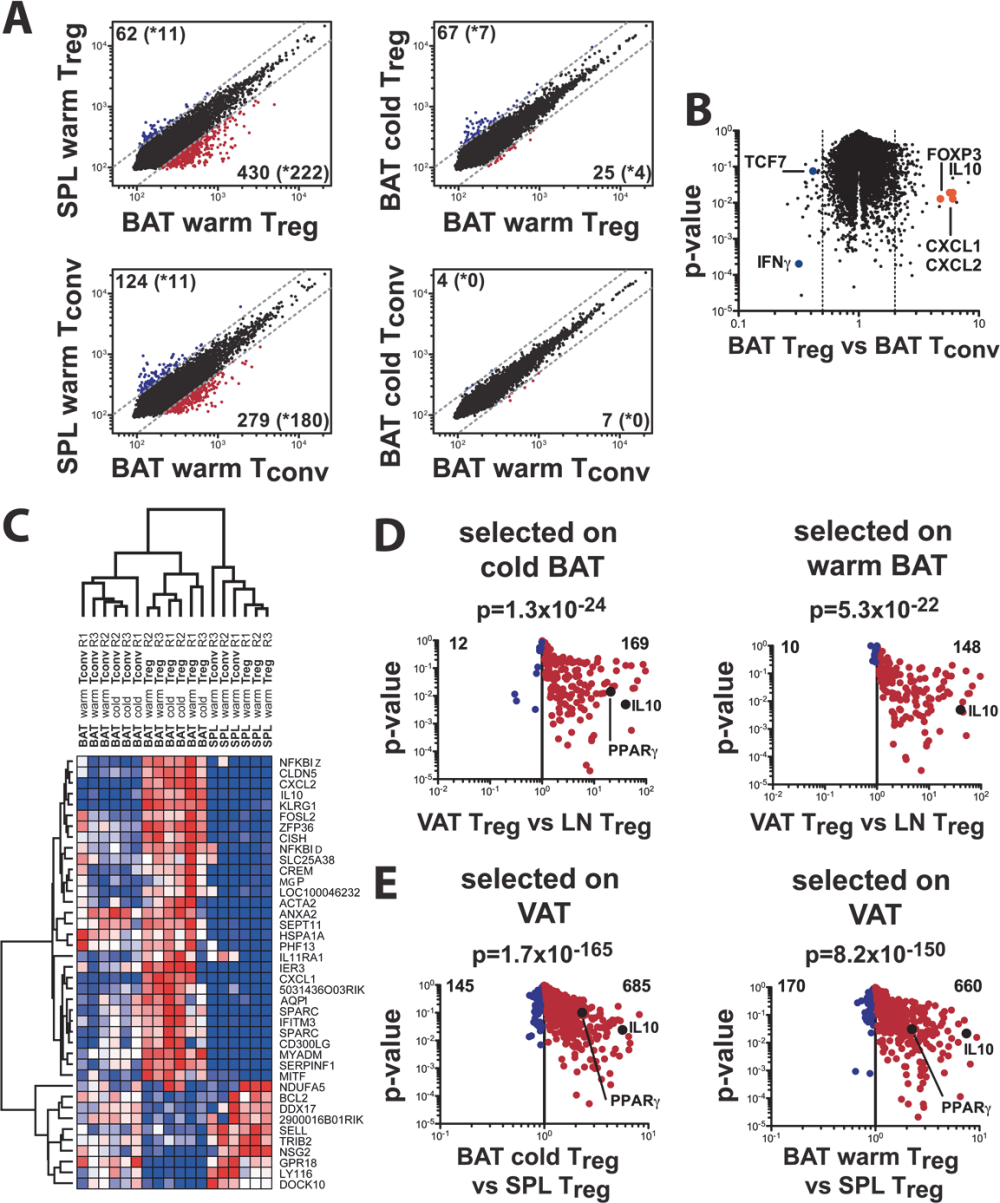
Genotypical comparison of T_reg_ and T_conv_ cells isolated from brown adipose tissue (BAT) and spleen tissue (SPL) in cold- and warm-conditioned animals generated with an Illumina Mouse Expression Array. (A) Gene expression profiles comparing T_reg_ (top) or T_conv_ (bottom) cell populations between spleen and adipose tissue samples isolated from warm-conditioned animals (left) or between cells isolated from cold vs warm-conditioned animals (right). Numbers indicate genes either up- or downregulated more than 2-fold (cut-off: dotted line), with the number of significantly different (p<0.05) genes shown in brackets with an asterisk. (B) Volcano plot comparing gene expression and significance values between T_reg_ and T_conv_ genes isolated from BAT in warm-conditioned animals. Key up- or downregulated genes in T_reg_ cells are annotated (Foxp3, Il10, Cxcl1/2, Tcf7, Ifng) and serve as quality control to the published consensus T_reg_-cell signature. (C) Hierarchical clustering of the top-30 upregulated genes and the top-10 downregulated genes in warm-conditioned brown adipose tissue T_reg_ cells versus spleen T_reg_ cells. (D) Comparison of BAT-T_reg_-specific genes with visceral adipose tissue (VAT)-specific genes. We first determined 430 genes to upregulated in BAT warm-conditioned T_reg_ cells, with 222 genes being significantly altered (p<0.05). We then overlaid BAT T_reg_-upregulated genes with VAT T_reg_ tissue specific expression gene data. 181 genes were matched between both microarary chips, with 169 genes also upregulated in VAT, and only 12 genes specific for BAT (left). The corresponding analysis of the 516 genes upregulated in cold BAT T_reg_ cells versus warm spleen T_reg_ cells revealed 194 genes to be significantly upregulated. 158 could be matched to VAT T_reg_-specific genes, of which 148 were VAT-specific, whereas only 10 were specific for BAT. P-values indicate the significance of overrepresentation of BAT T_reg_-specific genes in the VAT T_reg_ signature. (E) Comparison of VAT-T_reg_ specific genes on BAT warm (left) or BAT cold (right) gene signatures. Of 1839 genes specifically overexpressed in VAT T_reg_ cells, 1059 were statistically significantly (p<0.05) upregulated. Of these 1059 genes, 829 were also detectable in the BAT T_reg_ microarray. When comparing the VAT T_reg_ signature to warm BAT T_reg_ cells, 660 genes were overrepresented in VAT, whereas cold BAT T_reg_ cells show 685 genes to be overrepresented in VAT.

The overall importance of T_reg_ cells for WAT function prompted us to explore the molecular nature of BAT-associated T_reg_ cells. To this end, wild-type C57Bl6 mice were either acclimatized to 30°C or challenged with a 12°C cold exposure for 2 days. After the corresponding acclimatization periods, BAT depots as well as the spleens were collected and T_reg_ cells were sorted out of the corresponding tissue by fluorescence-associated cell sorting (FACS) followed by RNA purification and hybridization to an expression array. Microarray gene expression profiling revealed 430 genes to be more than twofold upregulated in BAT T_reg_ cells compared to splenic T_reg_ cells, with 222 genes significant between three replicates (Fig. 1A). Confirmation of lineage-specific genes in BAT T_reg_ versus BAT T_conv_ T cells included classic T_reg_ genes such as Foxp3 and cytotoxic T-lymphocyte-associated protein 4 (Ctla4) as over-represented and special AT-rich sequence binding protein 1 (Satb1) and transcription factor 7, T cell specific (Tcf7) as under-represented genes. In addition, chemokine (C-X-C motif) ligand (Cxcl) 1 and 2, and interleukin (IL) 10 were highly over-expressed in BAT T_reg_ cells (Fig. 1B). The top 30 significantly upregulated genes and bottom 10 downregulated genes in BAT T_reg_ cells were then subjected to unsupervised hierarchical clustering (Fig. 1C), which revealed a unique gene expression signature for BAT T_reg_ cells compared to splenic regulatory and conventional T (T_conv_) cells as well as BAT T_conv_ cells. We then compared the BAT T_reg_ gene signature to the VAT-specific gene signature [7], which stratified most of the genes identified in both microarrays to be specific for a general fat T_reg_ gene signature (Fig. 1D, E). However, a specific BAT T_reg_ gene signature could also be discerned, which was independent of the WAT T_reg_ signature (Table 1).

**Table 1:** Principal biological processes affected by T_reg_ cell depletion in BAT.

	Biological process	GO number	FDR (%)
Immune processes	Immune response	GO0006955	<0.001
	Inflammatory response	GO0006954	<0.001
	Innate immune response	GO0045087	0.048
	Adaptive immune response	GO0002250	0.151
			
	Chemotaxis	GO0006935	<0.001
	Leukocyte chemotaxis	GO0030595	0.008
	Cell migration	GO0016477	13.344
			
	Cell activation	GO0001775	0.057
	Myeloid leukocyte activation	GO0002274	<0.001
	Leukocyte activation	GO0045321	0.097
	T cell activation	GO0042110	0.194
	Lymphocyte activation	GO0046649	0.500
	Activation of immune response	GO0002253	1.459
			
	Cell proliferation	GO0008283	2.271
	Mononuclear cell proliferation	GO0032943	0.101
	T cell proliferation	GO0042098	0.113
	Lymphocyte proliferation	GO0046651	0.125
			
	Myeloid cell differentiation	GO0030099	0.051
	Leukocyte differentiation	GO0002521	4.297
	Lymphocyte differentiation	GO0030098	5.157
			
Cytokines	Cytokine and chemokine mediated signaling pathway	GO0019221	0.231
	Cytokine production	GO0001816	1.637
	Cytokine biosynthetic process	GO0042089	3.518
	Cytokine metabolic process	GO0042107	4.244
			
Metabolism	ATP metabolic process	GO0046034	11.567
	ATP biosynthetic process	GO0006754	8.646
	Phosphate transport	GO0006817	0.021
	ATP synthesis coupled proton transport	GO0015986	3.864

Next, we investigated whether differences exist between the gene expression profiles for cold- and warm-exposed T_reg_ cells. While the majority of genes was expressed at comparable levels in both cell types, 11 genes displayed significant gene expression changes in response to cold challenge (Fig. 1A), including coiled-coil-helix-coiled-coil-helix domain containing 10 (Chchd10), solute carrier protein family 52, member 3 (Slc52a3), protein tyrosine phosphatase, receptor type, S (Ptprs), RFT1 homolog (S. cerevisiae) (Rft1), dishevelled associated activator of morphogenesis 2 (Daam2), zinc finger protein 668 (Zfp668), stromal interaction molecule 1 (Stim1), family with sequence similarity 65, member B (Fam65b), RAS-like, family 11, member B (Rasl11b), centromere protein L (Cenpl), and polymerase (RNA) I polypeptide A (Polr1a) suggesting that this gene cluster represents a cold-specific T_reg_ cell signature in BAT.

### Cold activation of BAT requires T_reg_ cells for proper thermogenic gene expression

**Fig 2:**
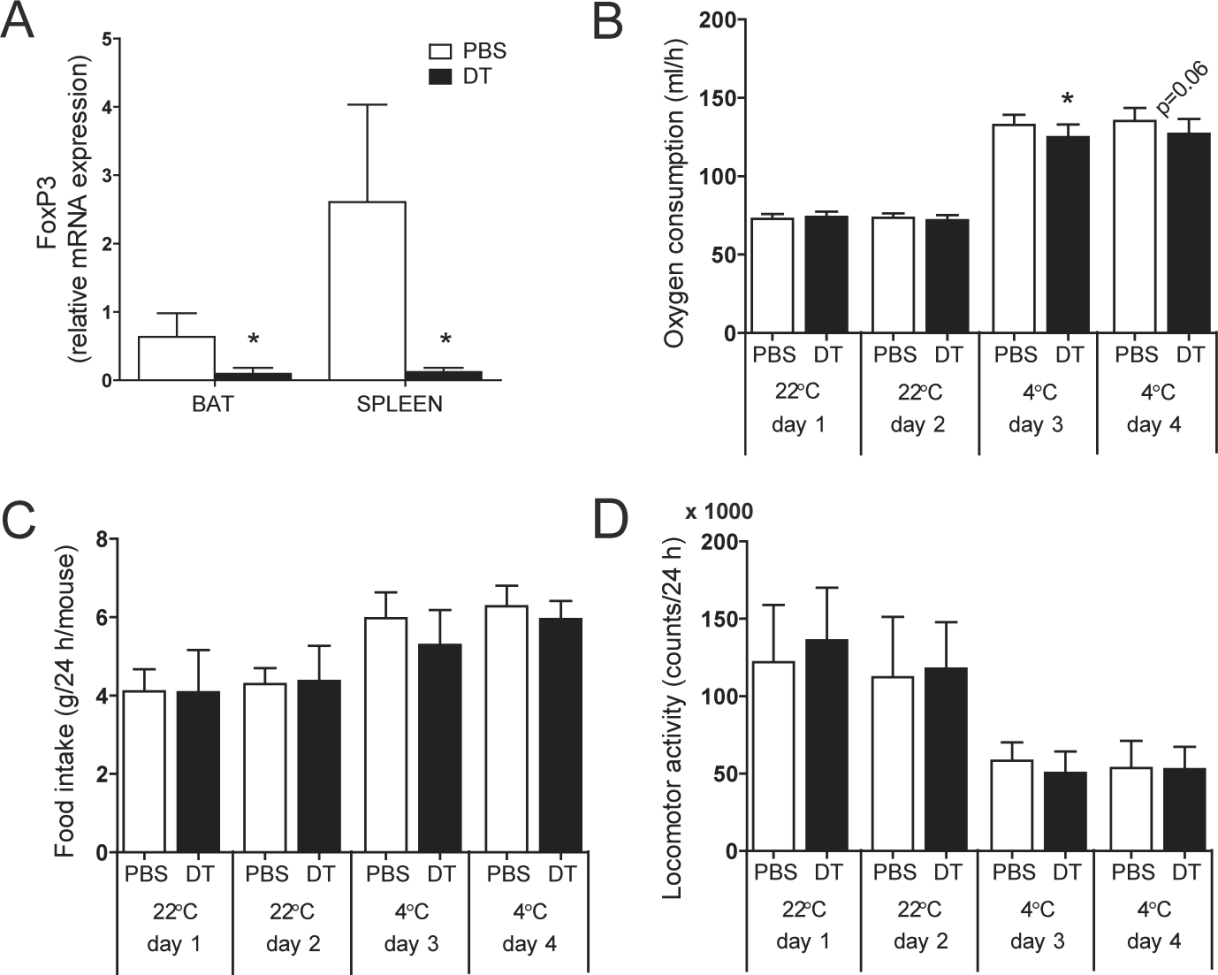
T_reg_ cell depletion and metabolic phenotyping. (A) Real-time RT-PCR analysis of FoxP3 expression in brown adipose tissue (BAT) and spleen in T_reg_ cell-proficient (PBS) and T_reg_ cell-deficient (DT) mice. (B) Oxygen consumption, (C) food intake and (D) locomotor activity during 4 consecutive days at 22°C and 4°C. Injections of vehicle (PBS) or diphtheria toxin (DT) were performed at day 2 and day 3. Values are mean ± SD (n = 9–10); *P<0.05 (Student’s t-test).

To next test the impact of T_reg_ cells on systemic energy expenditure under cold conditions, we performed deep metabolic phenotyping of T_reg_-proficient and T_reg_-deficient mice. Due to the lack of fat-specific T_reg_ targeting systems to date, we employed transgenic mice expressing the diphtheria toxin (DT) receptor under the control of Foxp3 gene regulatory elements [19]. Given the development of systemic immune dysfunction two weeks after DT administration in these animals [19], we restricted our analysis to the adaptation of systemic metabolism to an acute cold challenge and analyzed 48 h after T_reg_ depletion. Therefore, control littermates and T_reg_-depleted mice were challenged with an acute 4°C cold exposure after adaptation to room temperature (22°C). In line with previous studies [7], administration of DT to these animals resulted in the rapid systemic depletion of Foxp3-expressing cells (Fig. 2A).

While T_reg_ deficiency did not exert any effects on respiratory activity under room temperature conditions as demonstrated by unchanged oxygen consumption rates (Fig. 2B), depletion of T_reg_ cells significantly reduced oxygen consumption at cold conditions (Fig. 2B), while leaving food intake and locomotor activity unchanged (Fig. 2C, D).

**Fig 3:**
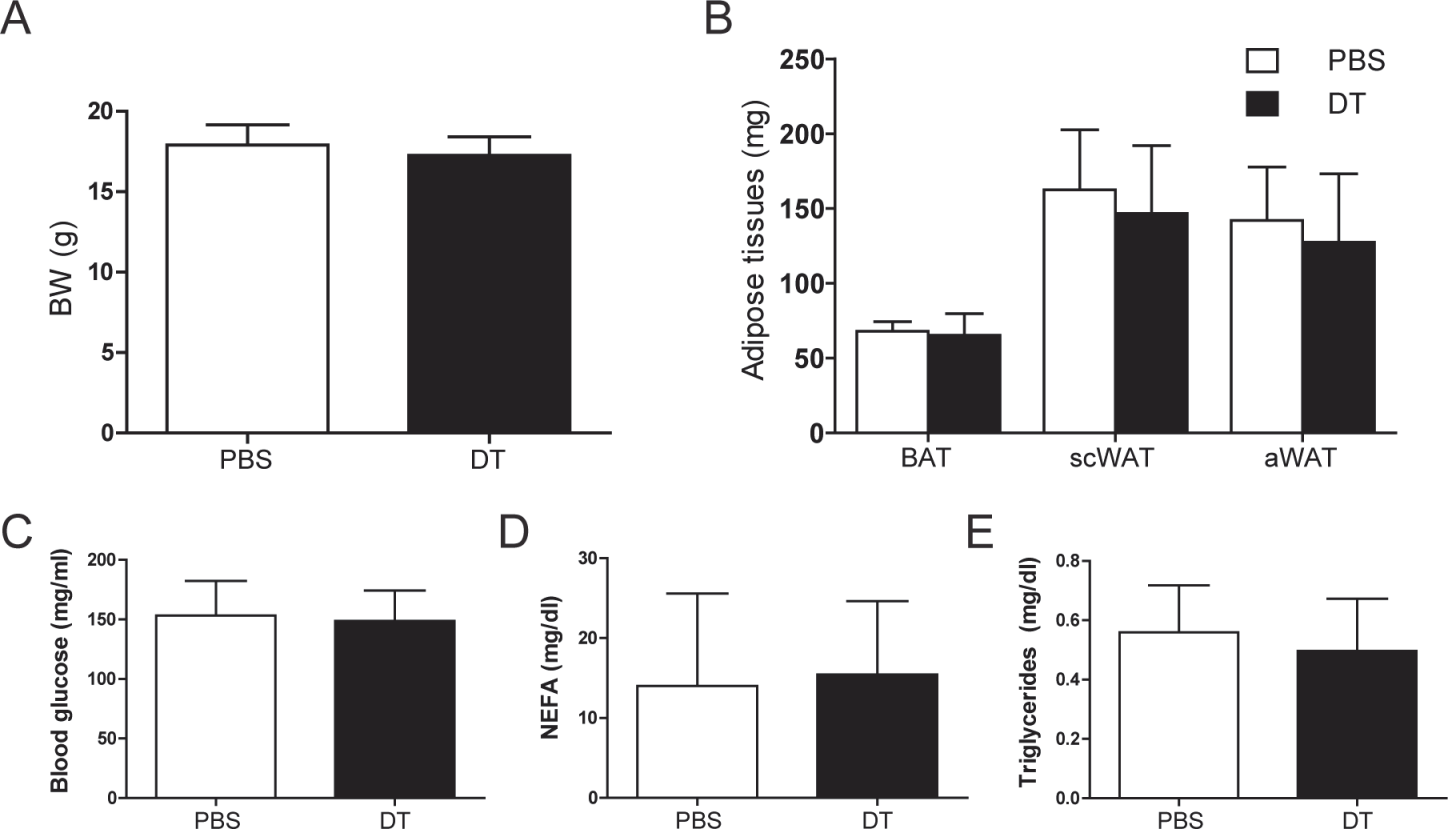
Physiological parameters. (A) Body weight (BW) and (B) adipose tissues weights of T_reg_ cell-proficient (PBS) and T_reg_ cell-deficient (DT) mice after cold exposure. BAT, brown adipose tissue; scWAT, subcutaneous white adipose tissue, aWAT, abdominal white adipose tissue. (C) Blood glucose, (D) serum non-estherified fatty acids (NEFA) and (E) serum triglycerides in PBS and DT mice. Values are mean ± SD (n = 9–10); *P<0.05 (Student’s t-test).

Of note, diphtheria toxin administration had no effect on oxygen consumption in wild-type animals (data not shonw), demonstrating the specificity of the observed effects. Also, body and adipose tissue weights and other metabolic parameters, including blood glucose, serum non-esterified fatty acids (NEFA) and triglycerides, remained unaffected by T_reg_ cell-depletion (Fig. 3A-E).

**Fig 4:**
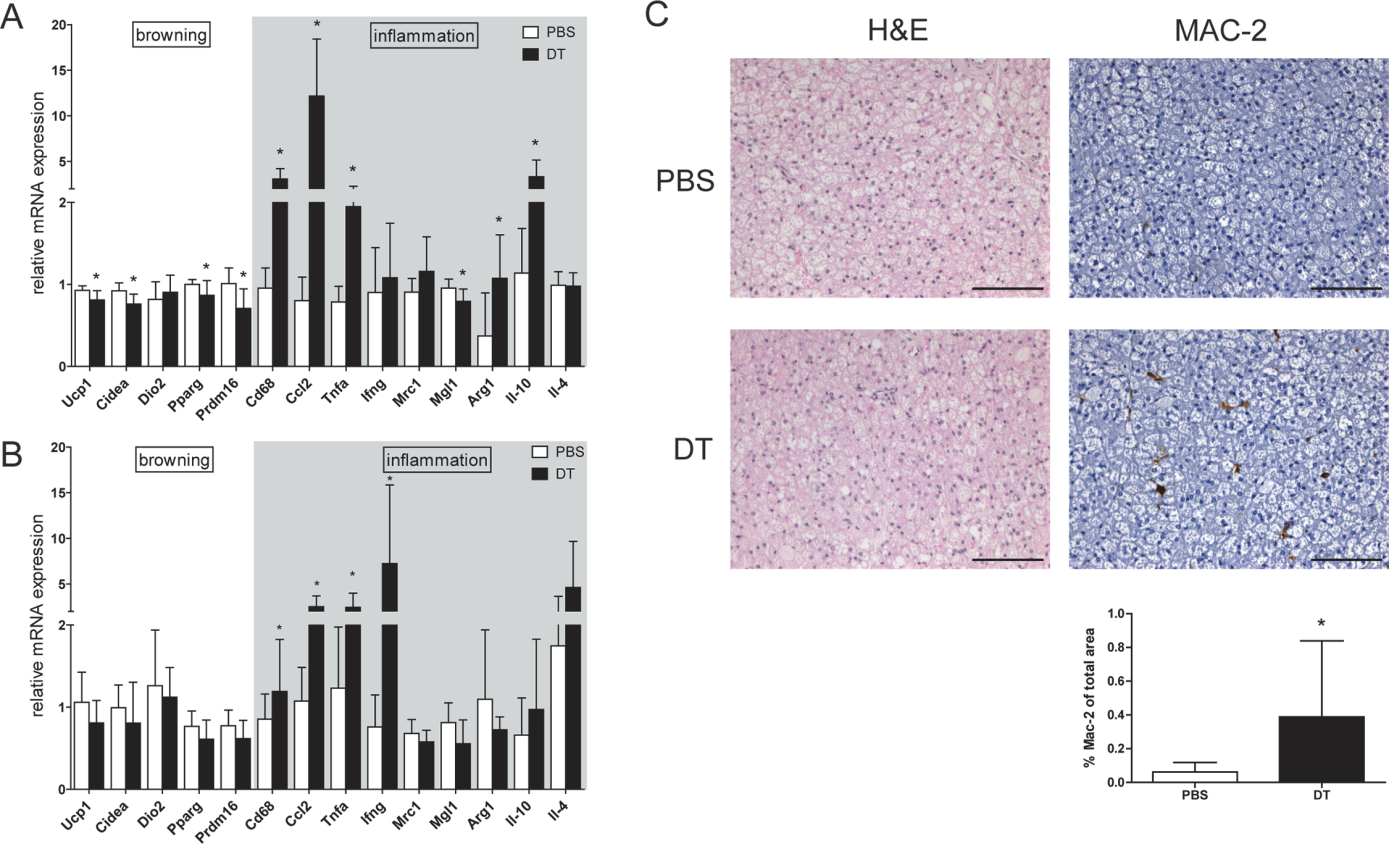
Inflammatory status of adipose tissue. Real-time RT-PCR analysis of (A) brown adipose tissue (BAT) and (B) subcutaneous white adipose tissue of T_reg_ cell-proficient (PBS) and T_reg_ cell-deficient (DT) mice after cold exposure. Ucp1, uncoupling protein 1; Cidea,cell death-inducing DNA fragmentation factor, alpha subunit-like effector A; Dio2,deiodinase, iodothyronine, type II; Pparg, peroxisome proliferator-activated receptor gamma; Prdm16, PR domain containing 16; Cd68, Cd68 antigen; Ccl2,chemokine (C-C motif) ligand 2; Tnfa, tumor necrosis factor alpha; Ifng, interferon, gamma; Mrc1, mannose receptor, C type 1; Mgl1, macrophage galactose-type C-type lectin 1; Arg1,arginase 1; Il-10, interleukin 10; Il-4, interleukin 4. Data are mean ± SD (n = 9–10); *p<0.05 (Student’s t-test). (C) Representative hematoxylin and eosin (H&E) staining (left) and immunohistochemical anti-MAC-2 staining (right; brown color) in BAT from PBS and DT mice. Scale bar 100 μm. Quantification of MAC-2 positive area (panel below MAC-2 staining) as a percentage of total area.

These data supported the hypothesis that T_reg_ cells may exert regulatory functions in BAT activation in response to cold stimulation. Consistent with impaired BAT activity, heat production per animal was decreased (S1 Fig.). Also, selective gene expression profiling revealed that particularly genes in the thermogenic gene program were downregulated in cold-exposed BAT upon T_reg_ cell deficiency (Fig. 4A), without affecting browning markers in subcutaneous WAT (Fig. 4B). Pro-inflammatory markers, including chemokine (C-C motif) ligand 2 (Ccl2) and tumor necrosis factor alpha (Tnfa), were found to be induced upon the lack of T_reg_ cell function. Along with these observations, T_reg_-deficient BAT displayed a substantial increase in macrophage infiltration (Fig. 4C).

### T_reg_ cells control distinct inflammatory and metabolic pathways in BAT

The data thus far indicated that suppressive T_reg_ cell function plays an important role in the execution of proper BAT function with implications for systemic energy expenditure, prompting us to analyze T_reg_-dependent molecular networks in BAT in more detail. To this end, we performed microarray analysis of cold-exposed T_reg_-deficient and -proficient BAT. Hierarchical clustering analysis showed a clear difference in transcription pattern between respective groups (S2 Fig.). It revealed that the absence of T_reg_ cells led to the marked upregulation of genes involved in immune processes. Most prominent among 107 significantly upregulated genes in T_reg_-deficient BAT were CC chemokine family members, namely Ccl2, -3, -6, -7 and -9. In parallel, expression of the macrophage marker Cd68 was upregulated. Increased infiltration of macrophages in BAT was indeed confirmed by immunohistochemical staining and its quantification for an additional macrophage marker MAC-2 (Fig. 4C). Moreover, high transcript levels of members of the Toll-like receptor family (Tlr7, -8, -13) were observed, suggesting activation of innate immune system in T_reg_ cell-deficient BAT. At the same time, a glycolytic enzyme aldolase A (Aldoa) and a glucose/fructose transporter solute carrier family 2 (facilitated glucose transporter), member 5 (Slc2a5) were downregulated in T_reg_-deficient BAT, which might indicate impaired glucose utilization. Manual inspection of the gene expression data for gene members of glycolytic pathway indeed demonstrated that expression of pyruvate dehydrogenase kinase, isoenzyme 1 (Pdk1), glucose-6-phosphatase, catalytic (G6pc), phosphoglycerate kinase 1 (Pgk1), hexokinase 1 (Hk) and phosphoglucomutase 3 (Pgm3) were decreased as well, although did not reach the stringent criteria for statistical significance.

Subsequent pathway analysis using Gene Set Enrichment Analysis (GSEA) software confirmed the hierarchical clustering and showed that the absence of T_reg_ cells led to substantial upregulation of immune processes, in particular of pro-inflammatory pathways involving cell differentiation, proliferation, trafficking and activation (Table 1). Furthermore, at the same time ATP metabolic processes were upregulated in T_reg_-deficient BAT (Table 1). Thus, T_reg_ cell ablation altered the activity in particular of pro-inflammatory pathways while at the same time controlling ATP synthesis-related genes and glycolysis overall suggesting that BAT-associated T_reg_ cells indeed impose a critical regulatory input into BAT macrophage activity as well as energy production.

## Discussion

In this study, we characterized the gene expression profile of BAT T_reg_ cells under two different temperature conditions and investigated the role of T_reg_ cells in BAT activation after cold challenge.

Recently, a unique population of T_reg_ cells with a distinct gene expression signature has been characterized in VAT [23] and its contribution to proper white adipose function has been described [11]. Given the opposing functions of white and brown adipose tissues, this prompted us to investigate T_reg_ cell population characteristics in BAT under different temperature conditions. The gene expression profile of BAT T_reg_ cells clearly differentiated them from spleen T_reg_ cells and from conventional BAT and spleen T cells. Direct comparison to the visceral T_reg_ cell expression profile revealed a close similarity of BAT and VAT T_reg_ cells, but also a BAT specific T_reg_ signature. Furthermore, we compared the T_reg_ cell expression profile from warm and cold temperature condition and identified 11 unique differentially expressed genes. The high correlation of BAT- and VAT-specific T_reg_ genes indicate common adipose tissue factors that induce the phenotype of fat resident T_reg_ cells. Indeed, Pparg, the central transcription factor involved in VAT T_reg_ cell accumulation and function [12], is also over-expressed in BAT T_reg_ cells, however, much less pronounced. The identification of a VAT independent but BAT-specific T_reg_ cell signature suggests that additional factors shape BAT T_reg_ cell phenotype and function.

Lack of T_reg_ cells [7] or their phenotypic change [24] in WAT upon high-fat feeding is associated with VAT dysfunction. In agreement with these findings, metabolic phenotyping after systemic T_reg_ cell depletion also indicated impaired BAT function, demonstrated by decreased oxygen consumption in T_reg_ depleted mice and prevention of the activation of thermogenic genes. Decreased thermogenesis is often translated into enhanced adiposity [25]; however, BW and fat pad weight after cold stimulation remained unchanged in T_reg_-proficient and T_reg_-deficient mice due to increased food intake, which most likely compensated for increased energy expenditure. Decreased thermogenesis in T_reg_-deficient mice did not affect adiposity, given only the short period of observation.

T_reg_ cell depletion potentiates tissue inflammation and influences number and activation state of macrophage and dendritic cells [19]. It has been shown that sustaining adaptive thermogenesis requires alternative activation of BAT-resident macrophages in cold-challenged mice [14], which was precluded upon T_reg_ cell depletion. Indeed, pro-inflammatory Tnfa and several members of CC chemokine family were among the most upregulated genes in cold-challenged T_reg_ cell depleted BAT. Therefore, impaired BAT function might be at least partly attributed to inability of alternative macrophage activation due to the loss of T_reg_ cell suppressive function and the markedly increased inflammatory state of the tissue, since inflammation can suppress induction of Ucp1 expression [26,27].

Cold exposure is associated with lipolysis stimulation and increased glucose uptake in BAT [28–30], where released fatty acids and glucose represent a metabolic fuel. In our setting, glucose transporter together with glycolytic pathway activation in T_reg_-deficient mice remained downregulated compared to control mice, indicating impaired fuel utilization. Moreover, ATP metabolic processes were upregulated in T_reg_-deficient BAT, suggesting that high inflammatory status of the tissue prevented heat production.

Our study defines the molecular signature of BAT-specific T_reg_ cells and also implies a functional role of T_reg_ cells in BAT function during cold exposure. Given the therapeutic potential of BAT activation for conditions of metabolic dysfunction, the modulatory role of BAT-associated T_reg_ cells may now provide clues for novel BAT-based therapies in obesity and type 2 diabetes.

## Supporting Information

S1 Fig Heat production. T_reg_ cell-proficient (PBS) and T_reg_ cell-deficient (DT) mice heat production during 4 consecutive days at 22°C and 4°C. Injections of vehicle (PBS) or diphtheria toxin (DT) were performed at day 2 and day 3. Values are mean ± SD (n = 9–10); *P<0.05.
(TIF)

S2 Fig Hierarchical clustering analysis of differentially expressed genes from T_reg_-proficient and -deficient BAT. Heat map showing all FDR-significant genes and genes with fold-change higher than twofold with nominal p-value below 0.01.
(TIF)

S1 Table BAT Treg-specific gene signature. Genes over-expressed in warm-conditioned BAT Treg cells compared to spleen Treg cells from the same mice were identified (2-fold up-regulated). These genes were then compared with visceral adipose tissue (VAT) Treg cells and selected for not-different expressed in visceral Treg vs lymph node Treg cells (less than 1.2-fold change). These 28 genes are listed with their differential expression values color-coded to indicate significance (p<0.05, red font color) and expression change between BAT and SPL tissue (two-fold induction, green font color). Genes with more than two-fold induction only in Treg BAT cells, but not in Tconv BAT cells, comprise a Treg-specific BAT gene signature.
(DOCX)
