# Supplementary figures and images for: Brown Adipose Tissue Harbors a Distinct Sub-Population of Regulatory T Cells
Source: PLoS One. 2015 Feb 25;10(2):e0118534. doi: 10.1371/journal.pone.0118534 (PMC4340926; doi:10.1371/journal.pone.0118534)

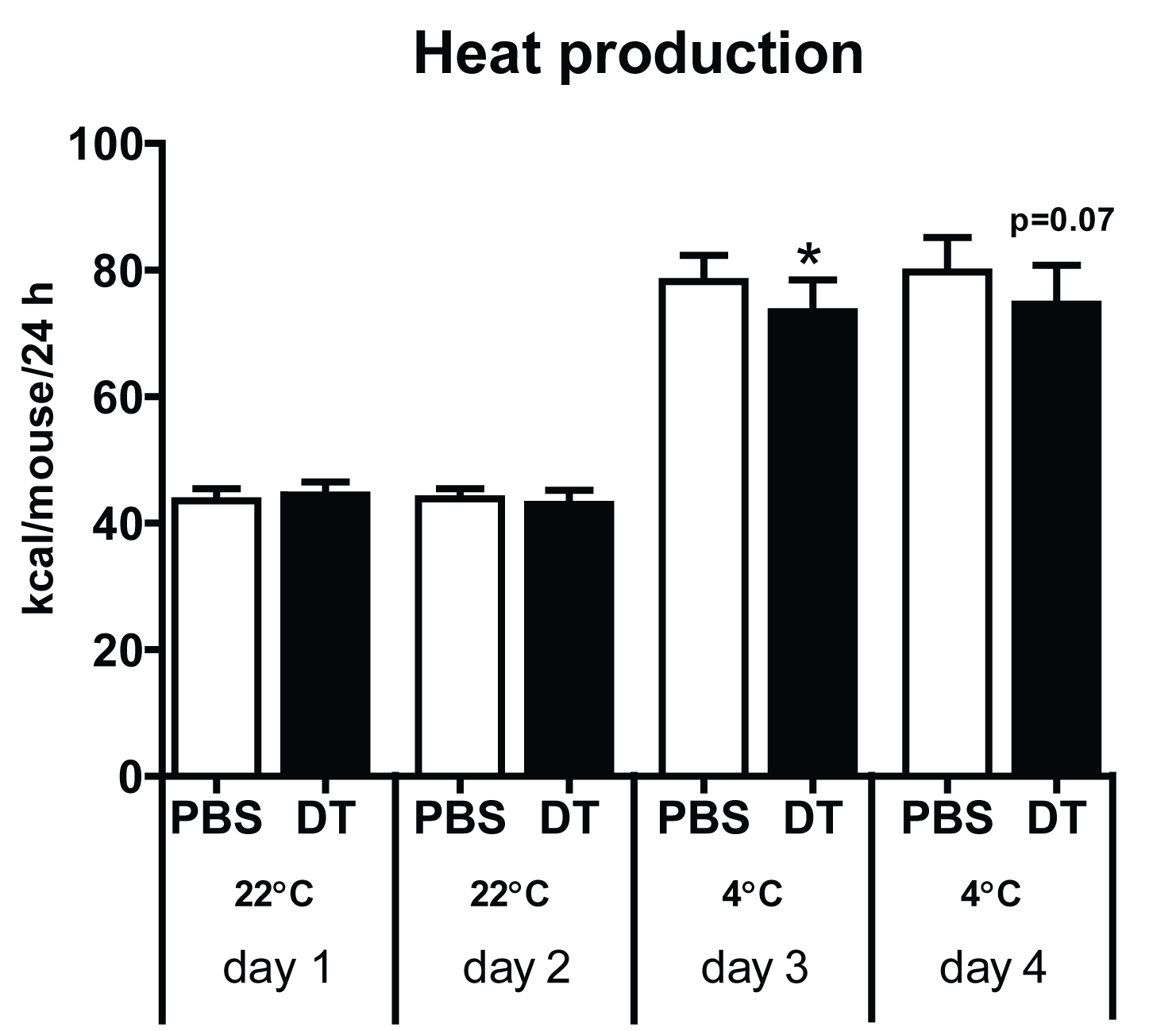

Supplement: S1 Fig — Treg cell-proficient (PBS) and Treg cell-deficient (DT) mice heat production during 4 consecutive days at 22°C and 4°C. Injections of vehicle (PBS) or diphtheria toxin (DT) were performed at day 2 and day 3. Values are mean ± SD (n = 9–10); *P<0.05. (TIF) [file pone.0118534.s001.tif]

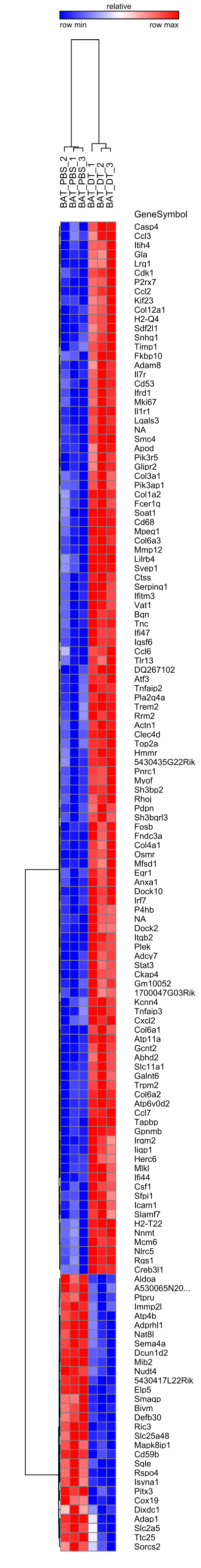

Supplement: S2 Fig — Heat map showing all FDR-significant genes and genes with fold-change higher than twofold with nominal p-value below 0.01. (TIF) [file pone.0118534.s002.tif]
