# Supplementary material for: Brown Adipose Tissue Harbors a Distinct Sub-Population of Regulatory T Cells
Source: PLoS One. 2015 Feb 25;10(2):e0118534. doi: 10.1371/journal.pone.0118534 (PMC4340926; doi:10.1371/journal.pone.0118534)
Supplement: S1 Table — Genes over-expressed in warm-conditioned BAT Treg cells compared to spleen Treg cells from the same mice were identified (2-fold up-regulated). These genes were then compared with visceral adipose tissue (VAT) Treg cells and selected for not-different expressed in visceral Treg vs lymph node Treg cells (less than 1.2-fold change). These 28 genes are listed with their differential expression values color-coded to indicate significance (p<0.05, red font color) and expression change between BAT and SPL tissue (two-fold induction, green font color). Genes with more than two-fold induction only in Treg BAT cells, but not in Tconv BAT cells, comprise a Treg-specific BAT gene signature. (DOCX) [file pone.0118534.s003.docx]

**Supplementary Table 1**

| **Gene name** | **BAT Treg vs SPL Treg** | **p-value** | **BAT Tconv vs SPL Tconv** | **p-value** |
| --- | --- | --- | --- | --- |
|  |  |  |  |  |
| 2310033F14RIK | 2.3072 | 0.0243 | 1.4077 | 0.1755 |
| AKAP9 | 2.9492 | 0.0322 | 1.6643 | 0.1459 |
| AKAP9 | 2.7805 | 0.0451 | 1.6276 | 0.1648 |
| B230380D07RIK | 3.0864 | 0.0156 | 1.0946 | 0.2256 |
| BC037034 | 3.5732 | 0.0342 | 0.8783 | 0.8550 |
| CHST2 | 2.4040 | 0.0143 | 2.9137 | 0.0344 |
| CXCR3 | 2.3766 | 0.0075 | 2.8601 | 0.0219 |
| D19WSU162E | 2.6084 | 0.0395 | 1.0395 | 0.9217 |
| ELK3 | 2.4153 | 0.0014 | 1.2409 | 0.4051 |
| GRINA | 2.0828 | 0.0243 | 1.2683 | 0.2372 |
| HCFC1 | 2.5447 | 0.0040 | 0.5565 | 0.0957 |
| HPRT1 | 2.2453 | 0.0163 | 1.8202 | 0.0330 |
| IFNG | 2.8331 | 0.0376 | 6.6587 | 0.0064 |
| IRF1 | 2.7469 | 0.0277 | 1.6627 | 0.2889 |
| LARS | 2.8904 | 0.0349 | 1.0905 | 0.6236 |
| MAPKAP1 | 2.2330 | 0.0064 | 1.9464 | 0.0557 |
| MTCH1 | 2.2702 | 0.0248 | 1.5886 | 0.1448 |
| PARP1 | 2.4296 | 0.0386 | 1.3416 | 0.3917 |
| PDE4D | 2.5652 | 0.0303 | 1.6329 | 0.3808 |
| PJA2 | 2.0018 | 0.0091 | 1.8963 | 0.0426 |
| RASGRP4 | 2.8086 | 0.0160 | 1.2669 | 0.6855 |
| RASL11B | 2.0390 | 0.0292 | 2.6238 | 0.1645 |
| RGS1 | 2.1414 | 0.0116 | 3.5543 | 0.0074 |
| RIN3 | 2.2300 | 0.0200 | 1.9723 | 0.0212 |
| SBF1 | 2.0407 | 0.0321 | 1.3023 | 0.1575 |
| SDC3 | 2.5299 | 0.0473 | 1.3140 | 0.1155 |
| SLC41A3 | 2.8518 | 0.0048 | 1.2701 | 0.5356 |
| SLC41A3 | 2.1879 | 0.0216 | 1.1987 | 0.5757 |
| SOCS3 | 2.3520 | 0.0204 | 1.8884 | 0.2683 |
| TALDO1 | 2.3524 | 0.0319 | 2.0468 | 0.0883 |

**Supplementary Table 1: BAT T_reg_-specific gene signature**. Genes over-expressed in warm-conditioned BAT T_reg_ cells compared to spleen T_reg_ cells from the same mice were identified (2-fold up-regulated). These genes were then compared with visceral adipose tissue (VAT) T_reg_ cells and selected for not-different expressed in visceral T_reg_ vs lymph node T_reg_ cells (less than 1.2-fold change). These 28 genes are listed with their differential expression values color-coded to indicate significance (p<0.05, red font color) and expression change between BAT and SPL tissue (two-fold induction, green font color). Genes with more than two-fold induction only in T_reg_ BAT cells, but not in T_conv_ BAT cells, comprise a T_reg_-specific BAT gene signature.
